# Complete genome sequence of *Lactobacillus pentosus* SLC13, isolated from mustard pickles, a potential probiotic strain with antimicrobial activity against foodborne pathogenic microorganisms

**DOI:** 10.1186/s13099-018-0228-y

**Published:** 2018-01-18

**Authors:** Min-Lang Huang, Jing-Yao Huang, Cheng-Yen Kao, Tony J. Fang

**Affiliations:** 1 Department of Food Science and Biotechnology, National Chung Hsing University, No. 250 Kuokuang Rd., Taichung, 40227 Taiwan; 2 Department of Medical Microbiology and Immunology, School of Medicine and Public Health, University of Wisconsin-Madison, Madison, WI 53706 USA; 3 Food Industry Research and Development Institute, Hsinchu, Taiwan

**Keywords:** Exopolysaccharide, Antimicrobial activity, *Lactobacillus*, PacBio, Probiotics

## Abstract

**Background:**

*Lactobacillus pentosus* SLC13 is a high exopolysaccharide (EPS)-producing strain with broad-spectrum antimicrobial activity and the ability to grow in simulated gastrointestinal conditions. SLC13 was isolated from mustard pickles in Taiwan for potential probiotic applications. To better understand the molecular base for its antimicrobial activity and high EPS production, entire genome of SLC13 was determined by PacBio SMRT sequencing.

**Results:**

*L. pentosus* SLC13 contains a genome with a 3,520,510-bp chromosome and a 62,498-bp plasmid. GC content of the complete genome was 46.5% and that of plasmid pSLC13 was 41.3%. Sequences were annotated at the RAST prokaryotic genome annotation server, and the results showed that the genome contained 3172 coding sequences and 82 RNA genes. Seventy-six protein-coding sequences were identified on the plasmid pSLC13. A plantaricin gene cluster, which is responsible for bacteriosins biosynthesis and could be associated with its broad-spectrum antimicrobial activity, was identified based on comparative genomic analysis. Two gene clusters involved in EPS production were also identified.

**Conclusion:**

This genomic sequence might contribute to a future application of this strain as probiotic in productive livestock potentially inhibiting competing and pathogenic organisms.

**Electronic supplementary material:**

The online version of this article (10.1186/s13099-018-0228-y) contains supplementary material, which is available to authorized users.

## Background

The beneficial effects of lactic acid bacteria (LAB) and its derivative exopolysaccharide (EPS), include the prevention and treatment of diarrheal disease, prevention of infections, antitumor activity, immunomodulation, prevention and treatment of allergies, and alleviation of lactose intolerance [1–6]. In our previous study, we isolated a high EPS-producing *Lactobacillus pentosus* strain, SLC13, from mustard pickles in Taiwan for potential probiotic applications [7]. SLC13 showed high resistance to bile salts, pH 3.0 PBS, and simulated gastrointestinal conditions. Moreover, the results of antimicrobial activity tests revealed that SLC13 showed high inhibitory activity against the growth of clinical important pathogens, including *Staphylococcus aureus, Streptococcus mutans, Enterococcus faecalis, Listeria monocytogenes* Scott A, *Yersinia enterocolitica, Escherichia coli* O157:H7, and *Salmonella enterica* sv. Typhimurium [7]. Here, the complete genome sequence of SLC13 was characterized to identify potential gene(s), which is (are) responsible for high EPS production or could be associated with its broad-spectrum antimicrobial activity.

## Methods

### Whole genome sequencing, assembly and annotation

The genomic DNA of SLC13 was extracted using the DNeasy Blood and Tissue Kit (QIAGEN, Germany), according to the manufacturer’s instructions. Total DNA was subjected to quality control by 2% agarose gel electrophoresis and quantified by a NanoDrop™ spectrophotometer. *L. pentosus* SLC13 was sequenced using the PacBio RS II platform (Pacific Biosciences, USA), and the reads were assembled using HGAP version 3.0. Sequences were further annotated at the RAST prokaryotic genome annotation server (http://rast.nmpdr.org/) [8].

### Analysis of antibiotic resistance genes, bacteriocin synthesis genes, and EPS-gene cluster

ResFinder databases (http://www.genomicepidemiology.org/) were used to find the antibiotic resistance genes present in the plasmid pSLC13. The first step of the bacteriocin identification workflow was created by merging the BACTIBASE databases using BLASTN [9] and BAGEL (class I, II and III) [10]. RAST server was used for identifying *eps* gene cluster by comparative genomic analysis.

### Quality assurance

The genomic DNA used for sequencing was isolated from a single colony of the SLC13. The *16S rRNA* gene was sequenced and BLAST was performed against the NCBI database. In addition, the raw read sequences were selected and assembled only when they satisfied the following criteria: minimum subread length, 500; minimum polymerase read quality, 0.8; and minimum polymerase read length, 100.

## Results and discussion

### General features

**Fig. 1:**
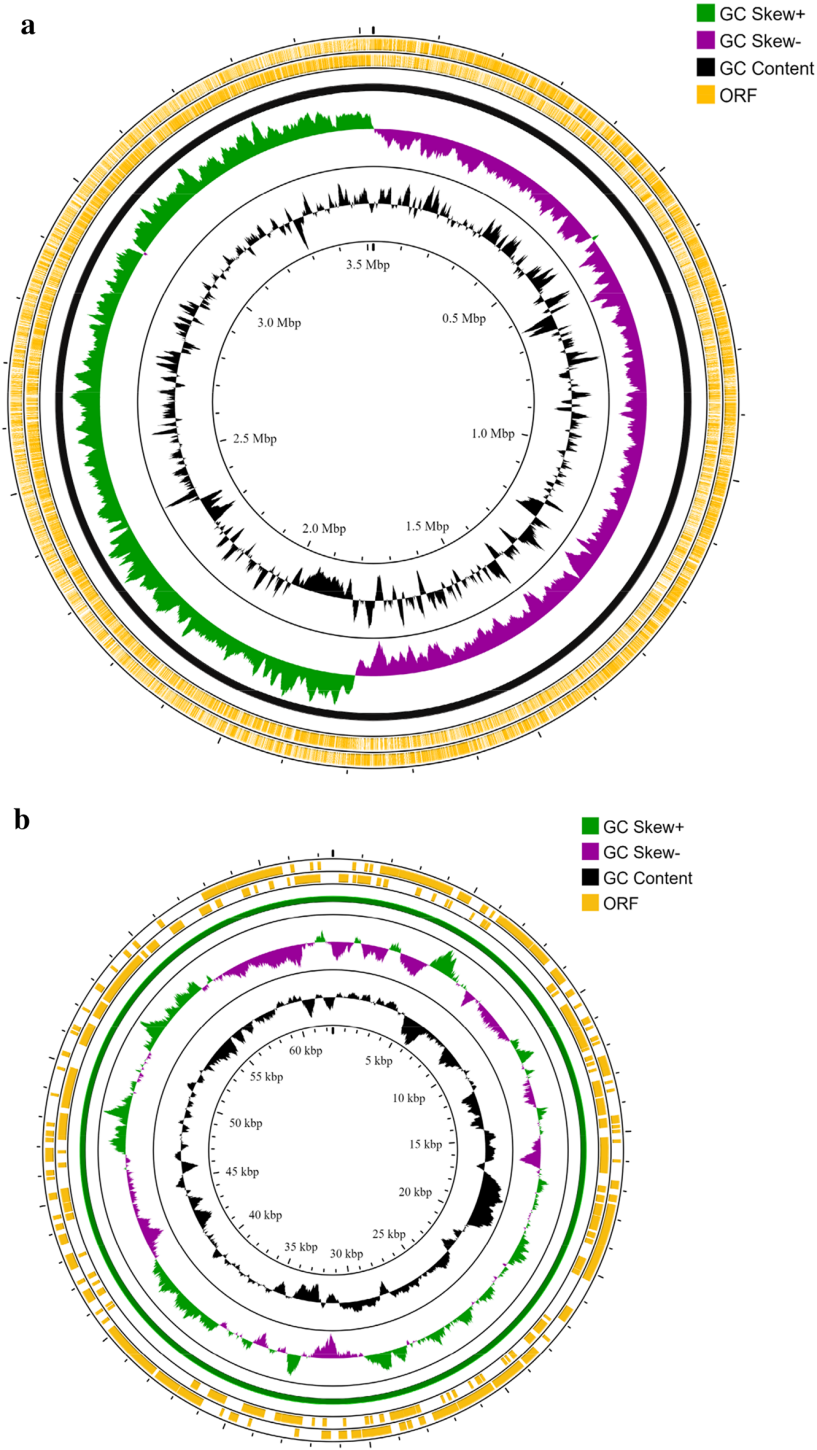
Circular genome map of *L. pentosus* SLC13. **a** Chromosome. **b** pSLC13. The scales indicate the location in Mbp, starting with the initial coding region. From the innermost circles, circle (1) GC content, plotted using a sliding window. Circle (2) shows the GC skew (G-C/G+C). The value is plotted as the deviation from the average GC skew of the entire sequence. Circle (3, 4) illustrate the coding sequences, 3 is backward strand, 4 is forward strand

*L. pentosus* SLC13 was sequenced using the PacBio RS II platform, generating a library containing 58,744 single reads with an average length of 3936 bp. Reads were assembled and returned two contigs with the head segment was almost identical to the tail segment, indicating the circular nature of the contigs. As shown in Fig. 1 and Table 1, the complete genome sequence of SLC13 was composed of a circular 3,520,510-bp chromosome and one 62,498-bp plasmid named as pSLC13. GC content of the complete genome was 46.5% and that of plasmid pSLC13 was 41.3%. The GC content and size of SLC13 chromosome was similar to other *Lactobacillus* strains, including *L. plantarum* GB-LP1 (3,040,388 bp, GC content: 44.9%) [11], *L. rhamnosus* CNCM I-3698 (2,966,480 bp, GC content: 46.69%) [12], *L. casei* BD-II (3,069,926 bp, GC content: 46.34%) [13], *L. pentosus* KCAI (3,418,159 bp, GC content: 46.4%) [14], but not *L. jojnsonii* FI9785 (1,755,993 bp, GC content: 34.49%) [15] (Table 1).

**Table 1:** Comparison of the features of *Lactobacillus* spp. genome and probiotic potential

Attributes	*Lactobacillus* strains					
Species	*L. pentosus*	*L. plantarum*	*L. rhamnosus*	*L. casei*	*L. pentosus*	*L. johnsonii*
Strain	SLC13	GB-LP1	CNCM I-3698	BD-II	KCA1	FI9785
Size (bp)	3,520,510	3,040,388	2,966,480	3,069,926	3,418,159	1,755,993
GC content (%)	46.5	44.9	46.69	46.34	46.4	34.49
Plasmid (no.)	1 (pSLC13: 62,498 bp)	–	–	1 (57,362 bp)	–	2 (p9785S: 3471 bp; p9785L: 25,652 bp)
RNAs (no.)	82	79	55	64	80	57
Coding sequences	3172 (76 in plasmids)	2899	2939	3139 (65 in plasmids)	2967	1710 (3 in p9785S; 25 in p9785L)
Resource (country)	Mustard pickles (Taiwan)	Traditional fermented food (Korea)	Goat rumen (UK)	Homemade koumiss (China)	Human vagina (Netherlands)	Poultry-derived (UK)
Probiotic potentials	Immune response, antibacterial activity	Immune response, suppression of pathogen growth, and antitoxin effects	Antagonistic activities against zoonotic pathogens	Reduce the blood lipid level and regulate the cardiovascular system	Produce biosurfactants, hydrogen peroxide (H_2_O_2_), and inhibit the growth of intestinal and urogenital pathogens	Immunomodulation, and competitive exclusion of pathogens

**Fig. 2:**
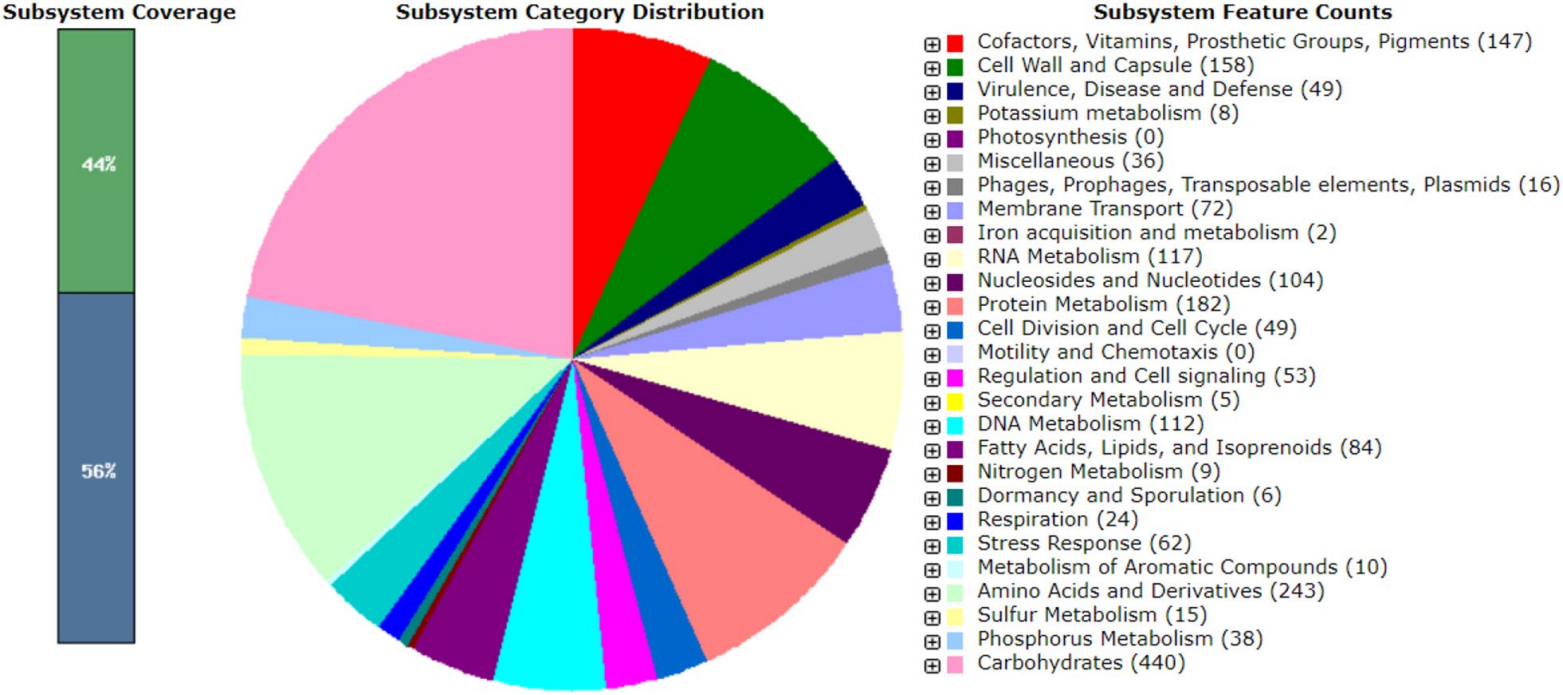
Subsystem distribution of *L. pentosus* SLC13 based on RAST annotation server. Out of 3172 coding sequences predicted by RAST server, the subsystem coverage is 44% which contributes to a total of 350 subsystems. The green bar of the subsystem coverage indicates the percentage of the proteins included in the subsystems while the blue bar refers to the percentage of the proteins that are not included in the subsystems

RAST annotation results showed that the genome contained 3172 coding sequences and 82 RNA genes (Table 1, Fig. 2). Moreover, all protein coding sequences were functionally annotated by RAST server in 350 subsystems (Fig. 2). Seventy-six protein-coding sequences were identified on the plasmids pSLC13. The sequence blast results revealed that pSLC13 showed high similarity to plasmid pLB1106-3 in *L. brevis* strain SRCM101106 (accession number: CP021675). In our previous study, we showed that SLC13 was resistant to penicillin G, cephalothin, cloxacillin, novobiocin, vancomycin, polymyxin B, rifampicin, tetercycline, kanamycin, gentamycin, neomycin, and streptomycin [7]. ResFinder databases were used to find the antibiotic resistance genes present in the plasmid pSLC13, and the results showed no antibiotic resistance genes were identified in pSLC13. Therefore, there is no further consideration for transmission of antibiotic resistant determinants by SLC13 as a candidate for probiotic development.

### Exopolysaccharide (*eps*) gene cluster identification

SLC 13 isolated from mustard pickles produced the largest amount of EPS (0.43 ± 0.04 g/l) among the 39 collected LAB isolates in our previous report [7]. To explore the gene cluster(s) that is (are) responsible for this phenotype, the comparative genomic analysis was performed by RAST server, and the genes that could be related to high-EPS biosynthesis were annotated in detail (Additional file 1: Figure S1). The genome of the SLC13 encoded 2 recognizable exopolysaccharide (*eps*) gene clusters. Comparison of the genome of *L. pentosus* SLC13 with that of strain *L. plantarum* WCFS1, indicated that the two EPS synthesis clusters assigned in SLC13 is highly conserved to the *L. plantarum* WCFS1 (Additional file 1: Figure S1). However, whether these two *eps* gene clusters responsible for high-EPS production in SLC13 is still unknown. Currently, Lee et al. reported that the abundance and sugar compositions of EPS diverse in three *L. plantarum* strains [16]. The diverse of sugar composition may affect its impact on probiotic-host interaction, for example, host cell adhesion [16]. Therefore, the composition of SLC13-EPS remains to be characterized in the future.

### Plantaricin (*pln*) gene cluster identification

A plantaricin (*pln*) gene cluster for bacteriocin synthesis in SLC13 identified by RAST server was shown in Additional file 1: Figure S2a. Further investigation was then conducted with BAGEL3, a web-based bacteriocin mining tool. The result from BAGEL3 showed a putative class II bacteriocin, Pediocin PA-1 immunity protein, was identified on SLC13 chromosome, as shown in Additional file 1: Figure S2b (gene start position, 2,037,423 on the chromosome; length, 112 amino acids). Pediocin PA-1 was previous identified in probiotic *Pediococcus acidilactici* with antimicrobial activity against *Listeria* spp., *Oenococcus oeni* and other wine bacteria [17, 18]. However, the antimicrobial activity of pediocin in SLC13 remains to be verified.

### Identification of genes coding for virulence factors

RAST annotation results showed that four virulence factors were found in the SLC13 chromosome, including heat shock protein 33, Jag, YidD, and YidC. Heat shock protein 33 is an adhesin important for adhesion and colonization of the *Streptococcus pyogenes* on epithelial cells [19]. Jag, YidD, and YidC are part of the *Mycobacterium* virulence operon with unclear function. Currently, Thakur et al. reported that the translocase YidC controls respiratory metabolism in *M. tuberculosis* and is required for the intracellular survival of *M. tuberculosis* in human macrophages [20]. A type II toxin-antitoxin system, mRNA interferase toxin, which is ubiquitous in *Lactobacillus* strains with the ability to inhibit *E. coli* growth, was found in pSLC13 [21]. However, the function of virulence factors identified in SLC13 remains to be determined.

Together, the complete genome sequence of SLC13 allows us to better understand molecular basis of its antimicrobial activity, high EPS production and probiotic potentials. Future studies are needed to verify the probiotic properties and safety of this strain for its industry application.

## Additional file

**Additional file 1: Figure S1.** Genetic organization of *eps* gene cluster of *L. pentosus* SLC13 and *L. plantarum* WCFS1. The graphic is centered on the focus gene (tyrosine-protein kinase transmembrane modulator, *epsC*), which is red and numbered 1. Sets of genes with similar sequence are grouped with the same number and color. Genes whose relative position is conserved in at least four chromosome loci are functionally coupled and share gray background boxes. Gene number 2, UDP-glucose 4-epimerase; number 3, tyrosine-protein kinase *epsD*; number 4, manganese-dependent protein-tyrosine phosphatase; number 5, undecaprenyl-phosphate galactosephosphotransferas; number 6, excinuclease ABC subunit C; number 7, exopolysaccharide biosynthesis glycosyltransferase *epsF*; number 8, integrase/recombinase (putative); number 9, glutamine ABC transporter, ATP-binding protein; number 10, dTDP-4-dehydrorhamnose reductase. **Figure S2.** Genetic organization of genes for bacteriocins synthesis. (A). Genetic organization of *pln* gene cluster of *L. pentosus* SLC13 and *L. plantarum* WCFS1. The graphic is centered on the focus gene (*plnP*), which is red and numbered 1. Sets of genes with similar sequence are grouped with the same number and color. Gene number 2, Three-component quorum-sensing regulatory system, sensor histidine kinase; number 3, Three-component quorum-sensing regulatory system, response regulator; number 4, *plnL*; number 5, Na(+)/H(+) antiporter; number 6, Branched-chain amino acid transport system carrier protein. (B). Genetic organization of putative class II bacteriocin on SLC13 chromosome predicted by BAGEL3 server. The gene for putative bacteriocin is shown in green.

## Abbreviations

EPS: exopolysaccharide
LAB: lactic acid bacteria
