# Supplementary material for: Complete genome sequence of Lactobacillus pentosus SLC13, isolated from mustard pickles, a potential probiotic strain with antimicrobial activity against foodborne pathogenic microorganisms
Source: Gut Pathog. 2018 Jan 18;10:1. doi: 10.1186/s13099-018-0228-y (PMC5774169; doi:10.1186/s13099-018-0228-y)
Supplement: Supplementary file 1 — Additional file 1: Figure S1. Genetic organization of eps gene cluster of L. pentosus SLC13 and L. plantarum WCFS1. The graphic is centered on the focus gene (tyrosine-protein kinase transmembrane modulator, epsC), which is red and numbered 1. Sets of genes with similar sequence are grouped with the same number and color. Genes whose relative position is conserved in at least four chromosome loci are functionally coupled and share gray background boxes. Gene number 2, UDP-glucose 4-epimerase; number 3, tyrosine-protein kinase epsD; number 4, manganese-dependent protein-tyrosine phosphatase; number 5, undecaprenyl-phosphate galactosephosphotransferas; number 6, excinuclease ABC subunit C; number 7, exopolysaccharide biosynthesis glycosyltransferase epsF; number 8, integrase/recombinase (putative); number 9, glutamine ABC transporter, ATP-binding protein; number 10, dTDP-4-dehydrorhamnose reductase. Figure S2. Genetic organization of genes for bacteriocins synthesis. (A). Genetic organization of pln gene cluster of L. pentosus SLC13 and L. plantarum WCFS1. The graphic is centered on the focus gene (plnP), which is red and numbered 1. Sets of genes with similar sequence are grouped with the same number and color. Gene number 2, Three-component quorum-sensing regulatory system, sensor histidine kinase; number 3, Three-component quorum-sensing regulatory system, response regulator; number 4, plnL; number 5, Na(+)/H(+) antiporter; number 6, Branched-chain amino acid transport system carrier protein. (B). Genetic organization of putative class II bacteriocin on SLC13 chromosome predicted by BAGEL3 server. The gene for putative bacteriocin is shown in green. [file 13099_2018_228_MOESM1_ESM.doc]

**Additional data**

**Fig. S1. Genetic organization of *eps* gene cluster of *L. pentosus* SLC13 and *L. plantarum* WCFS1.** The graphic is centered on the focus gene (tyrosine-protein kinase transmembrane modulator, *epsC*), which is red and numbered 1. Sets of genes with similar sequence are grouped with the same number and color. Genes whose relative position is conserved in at least four chromosome loci are functionally coupled and share gray background boxes. Gene number 2, UDP-glucose 4-epimerase; number 3, tyrosine-protein kinase *epsD*; number 4, manganese-dependent protein-tyrosine phosphatase; number 5, undecaprenyl-phosphate galactosephosphotransferas; number 6, excinuclease ABC subunit C; number 7, exopolysaccharide biosynthesis glycosyltransferase *epsF*; number 8, integrase/recombinase (putative); number 9, glutamine ABC transporter, ATP-binding protein; number 10, dTDP-4-dehydrorhamnose reductase.

**Fig. S2. Genetic organization of genes for bacteriocins synthesis.** (A). Genetic organization of *pln* gene cluster of *L. pentosus* SLC13 and *L. plantarum* WCFS1. The graphic is centered on the focus gene (*plnP*), which is red and numbered 1. Sets of genes with similar sequence are grouped with the same number and color. Gene number 2, Three-component quorum-sensing regulatory system, sensor histidine kinase; number 3, Three-component quorum-sensing regulatory system, response regulator; number 4, *plnL*; number 5, Na(+)/H(+) antiporter; number 6, Branched-chain amino acid transport system carrier protein. (B). Genetic organization of putative class II bacteriocin on SLC13 chromosome predicted by BAGEL3 server. The gene for putative bacteriocin is shown in green.

**Figure 1.**

**
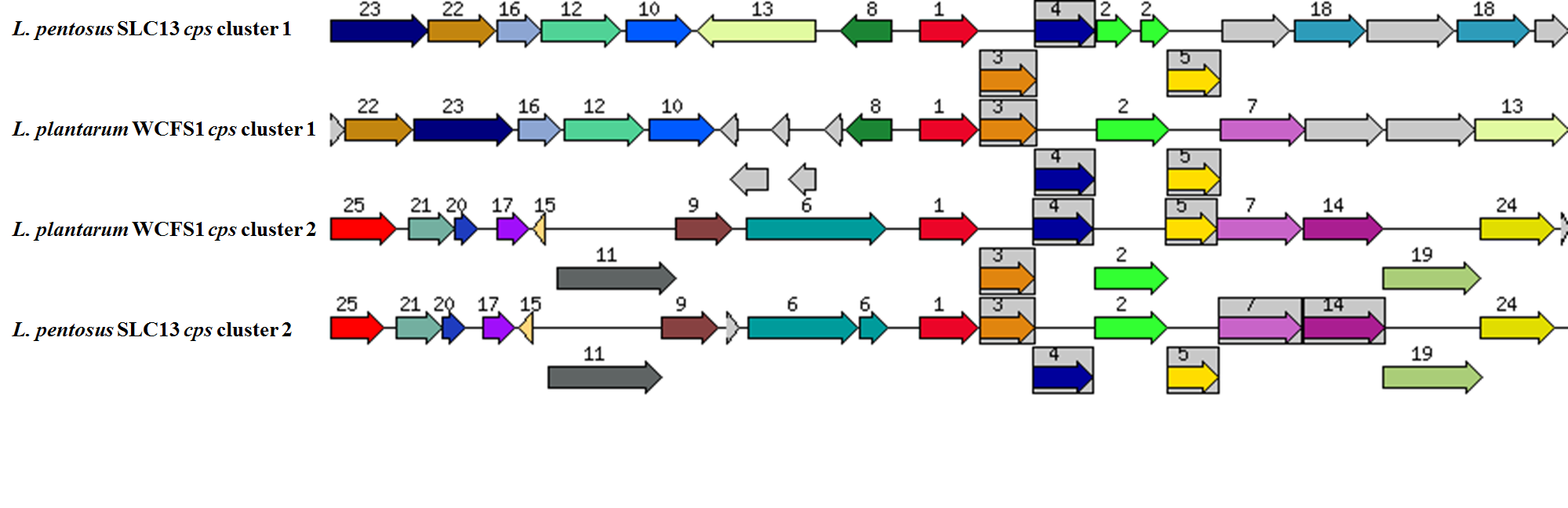
**

**Figure 2.**

**(A).**

**
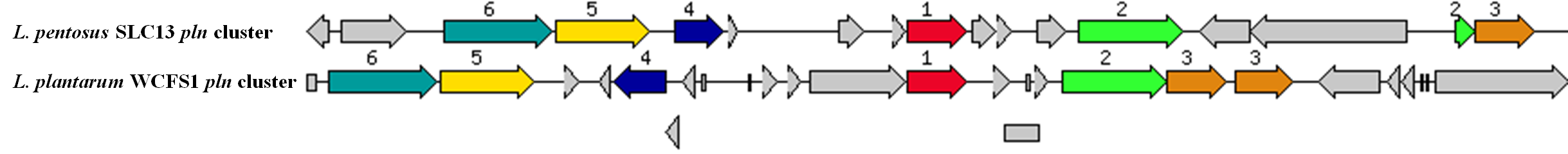
**

**(B).**

**
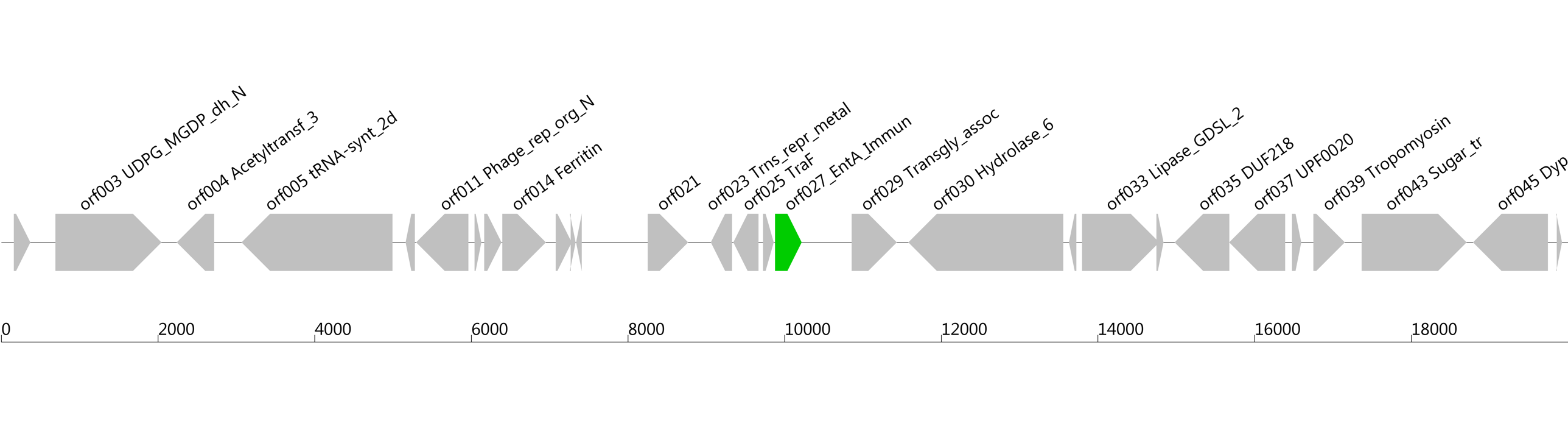
**
